# An evaluation of human protein-protein interaction data in the public domain

**DOI:** 10.1186/1471-2105-7-S5-S19

**Published:** 2006-12-18

**Authors:** Suresh Mathivanan, Balamurugan Periaswamy, TKB Gandhi, Kumaran Kandasamy, Shubha Suresh, Riaz Mohmood, YL Ramachandra, Akhilesh Pandey

**Affiliations:** 1 Institute of Bioinformatics, International Technology Park, Bangalore, India; 2 McKusick-Nathans Institute of Genetic Medicine and the Departments of Biological Chemistry, Pathology and Oncology, Johns Hopkins University, Baltimore, MD 21205, USA; 3 Department of Biotechnology, Kuvempu University, Shankaraghatta, Karnataka, India

## Abstract

**Background:**

Protein-protein interaction (PPI) databases have become a major resource for investigating biological networks and pathways in cells. A number of publicly available repositories for human PPIs are currently available. Each of these databases has their own unique features with a large variation in the type and depth of their annotations.

**Results:**

We analyzed the major publicly available primary databases that contain literature curated PPI information for human proteins. This included BIND, DIP, HPRD, IntAct, MINT, MIPS, PDZBase and Reactome databases. The number of binary non-redundant human PPIs ranged from 101 in PDZBase and 346 in MIPS to 11,367 in MINT and 36,617 in HPRD. The number of genes annotated with at least one interactor was 9,427 in HPRD, 4,975 in MINT, 4,614 in IntAct, 3,887 in BIND and <1,000 in the remaining databases. The number of literature citations for the PPIs included in the databases was 43,634 in HPRD, 11,480 in MINT, 10,331 in IntAct, 8,020 in BIND and <2,100 in the remaining databases.

**Conclusion:**

Given the importance of PPIs, we suggest that submission of PPIs to repositories be made mandatory by scientific journals at the time of manuscript submission as this will minimize annotation errors, promote standardization and help keep the information up to date. We hope that our analysis will help guide biomedical scientists in selecting the most appropriate database for their needs especially in light of the dramatic differences in their content.

## Background

Protein-protein interactions (PPI) are essential for almost all cellular functions. Proteins seldom carry out their function in isolation; rather, they operate through a number of interactions with other biomolecules. Experimental elucidation and computational analysis of the complex networks formed by individual protein-protein interactions (PPIs) is one of the major challenges in the post-genomic era. PPI databases have thus become valuable resources for the systematic analysis of the molecular networks of a cell [1,2]. With the accumulation of PPIs from high-throughput experiments, it is increasingly important to store such data for easy retrieval and analysis [3]. Several databases have compiled protein interactions based on manual curation of the scientific literature, automated text mining of articles or computational predictions. In this review, various features of nine different databases are evaluated, including compliance with emerging data standards such as proteomics standards initiative – molecular interaction (PSI-MI) format [4] and BioPAX [5], which define a unified framework for sharing PPI and pathway information, respectively.

### Human protein-protein interaction databases

Protein interaction repositories can be broadly classified into 2 types based on their content: i) Those containing interactions supported by experimental evidence, or, ii) Those containing interactions derived from *in silico *predictions alone, or, mixed together with experimentally derived PPIs. Here, we evaluate only those databases that exclusively contain experimentally derived PPI data in humans.

Curated literature based repositories have two major mechanisms of incorporating PPIs supported by experimental validation: i) curation by biologists from the literature, or, ii) direct deposit of the experimentally derived PPIs prior to publication by an investigator. Currently, the majority of PPIs in most databases are from curation of the literature. If all scientific journals mandated that PPIs be submitted to repositories as a requirement for publication (as is currently the case with nucleotide sequences), the databases would not only become more comprehensive but perhaps also contain fewer annotation errors. Below, we will briefly describe salient features of nine major PPI databases.

#### Human Protein Reference Database (HPRD)

HPRD contains annotations pertaining to human proteins based on experimental evidence from the literature [6,7]. This includes PPIs as well as information about post-translational modifications, subcellular localization, protein domain architecture, tissue expression and association with human diseases. In addition to interactions of proteins with other proteins, HPRD also reports interactions of proteins with nucleic acids and small molecules. The PPI data is sub classified as binary or complex interactions based on topology and the number of participants. Binary PPIs are direct interactions between two proteins while complexes represent interactions with more than 2 participants and the topology of interaction is unknown. Relevant publications are cited for each interaction. The type of experiment is also indicated as *in vivo *(e.g. coimmunoprecipitation), *in vitro *(e.g. GST pull-down assays) or yeast two-hybrid. Information about post-translational modifications includes the residue of modification, type of experiment and the upstream enzyme. These modifications can be viewed alongside the protein domain architecture. Each protein is linked to a genome browser, GenProt Viewer [8], which allows protein and transcript information to be visualized in the context of the relevant gene. HPRD is also linked to a compendium of signal transduction pathways, NetPath [9], which is freely available in several different formats. This database includes a tool called *PhosphoMotif Finder*, which reports the presence of any of over 320 phosphorylation-based motifs curated from the literature in a protein of interest. HPRD also incorporates a new feature, Protein Distributed Annotation System (PDAS) which allows researchers to contribute and share their data with the rest of the community.  All interaction information can be downloaded from the website either in PSI-MI format or as tab delimited files.

#### IntAct

The PPI information in the IntAct database includes a brief description of the interaction, experimental method and the literature citation of human proteins as well as proteins derived from several other species [10,11]. Whenever possible, PPI information is isoform specific. The database can be accessed by either a basic or advanced search. The latter provides the user with additional querying options such as experimental method or controlled vocabulary terms listed in PSI-MI. IntAct also has a tool which predicts best baits for pull-down experiments in humans by prioritizing the proteins which have the highest likelihood of being highly connected, or hubs, based on the available data within IntAct for various species – this is termed Pay-As-You-Go algorithm. Additional software developed as part of the IntAct project includes HierarchView, which depicts interaction networks as 2-dimensional graphs and highlights nodes based on a GO category specified by the user (e.g. cellular component).

#### Molecular INTeraction database (MINT)

MINT is a repository of experimentally verified protein interactions with special emphasis on mammalian interactions [12,13]. It also features interactions involving non-protein entities such as promoter regions and mRNA transcripts. PPI information includes binary and complex interactions and is isoform specific. Each interaction is given a confidence score based on the number of interactions and type of experiment and the number of citations provided for each interaction. The interactors can be viewed graphically using the 'MINT Viewer,' which permits users to view interactors as a network, and to manipulate it such that only the proteins of interest are shown. Users can expand the network by dragging individual interactors, select and visualize PPIs based on confidence scores, and they can also export the data in flat files, PSI-MI format or to Osprey, a system developed for visualizing and manipulating network data [14]. The interaction data are displayed along with the corresponding Swiss-Prot annotation. Proteins with a role in genetic diseases (according to OMIM (Online Mendelian Inheritance in Man)) are further highlighted. MINT features a separate annotation of human PPIs called HomoMINT, which includes in addition to literature derived data information from other organisms mapped to their human orthologs.

#### Database of Interacting Proteins (DIP)

PPI data stored in DIP were obtained through manual curation of the scientific literature and include direct and complex interactions [15,16]. The JDIP is a Java application based visualization tool; it provides a graphical representation of interactions. New high-throughput experimental and predicted PPI data can be evaluated through other services provided by DIP such as Paralogous Verification Method (PVM), Expression Profile Reliability (EPR) [17] and Domain Pair Verification (DPV) [18]. PVM validates interacting pairs by showing the existence of paralogous interactions; EPR validates comparison based on common expression profiles of interactors and DPV validates through domain-domain interaction preferences. Other satellite projects, Live-DIP and DLRP, use the DIP database for accessing the interactions. Live-DIP annotates proteins under different physiological conditions [19] whereas DLRP annotates protein-ligand and protein-receptor pairs known to interact with each other [20].

#### MIPS Database

MIPS database consists of mammalian interaction data manually curated from the literature [21,22], and includes experiment type, description of the interaction and binding regions of interacting partners (where available). Data from mass spectrometry and yeast two-hybrid studies are not included. PPIs can be queried based on interaction partners, experimental method, and functional aspects of the PPIs. The results can be retrieved in 2 formats – long and short. The long format details the interaction, including reference, experimental details, binding sites for each protein and a short comment on each interaction, its functional significance or the immediate outcome of the interaction. The short format is restricted to listing the interacting proteins. Both formats are also linked to visualization tools. Each protein is further linked to the corresponding annotation in the mouse PEDANT genome database developed by the same group; which contains pre-computed bioinformatics analyses of publicly available genomes [23].

#### Alliance For Cellular Signaling (AfCS)

The AfCS is a multidisciplinary, multi-institutional consortium that studies cellular signaling [24,25]. "Molecule Pages" in the AfCS database provide qualitative and quantitative information on signaling molecules (mostly murine) and their interactions; – these include results of experiments carried out by the Alliance in addition to literature-derived data. The molecule pages contain automated as well as author-entered data. The former integrate DNA/protein sequence information and structural details along with basic biophysical and biochemical properties from external databases, whereas the latter consist of data manually curated from the literature. This is further assessed by AfCS-appointed editorial board members and anonymously peer-reviewed in a process established by the Nature Publishing Group. The curated data includes a textual description of protein function, regulation of activity, subcellular localization, major sites of expression, splice variants and phenotype of knockout animals. The interaction data are derived from murine proteins, or, if they are from other species, the interaction is mapped to the corresponding mouse orthologs. For some proteins, the annotations include descriptions of signaling molecules under different physiological conditions termed 'states' (e.g. binding of a phosphorylated protein with another protein). A number of signaling pathway maps are also available in this database. We have not considered this database in our comparison mainly because of its focus on murine, and not human, proteins.

#### Biomolecular Interaction Network Database (BIND)

BIND is a database of biomolecular associations that are classified into 3 categories, binary molecular interactions, molecular complexes and pathways [26,27]. In BIND, a molecular complex is a collection of two or more molecules that associate to form a functional unit in a cell. These records are supplemented with additional information such as complex topology and the number of subunits involved in the interaction. Pathways are a collection of two or more interactions that occur in a defined sequence within a living system; currently 8 pathways have been annotated. Data pertaining to 1473 organisms is available in BIND. Information on molecular associations is obtained from the literature. The majority of the interactions in BIND are PPIs although it includes some interactions with nucleic acids and small molecules as well. The function of proteins is depicted using ontoglyphs, a series of symbolic characters representing a high-level summary of Gene Ontology (GO) information, and, proteoglyphs, symbols used to represent the structural and binding properties of proteins at the level of conserved domains. Data in BIND can be queried using various database identifiers or by a BLAST search. BIND also stores biomolecular interactions for several other species. For yeast high-throughput PPI datasets, BIND provides a confidence measure based on text mining of publications, existence of homologous interactions, common and related GO annotations, domain composition and phenotypic profiling for the evaluation. The data can be downloaded in flat file and PSI-MI formats and the pathways can be exported to 'sif' format which allows visualization by Cytoscape, a software tool developed for visualization and manipulation of pathway data [28]. BIND offers a Standard Object Access Protocol (SOAP) interface for those who wish to access the data from third-party software. BIND also has data imports from FlyBase, MIPS, MGI etc. and entries can be queried through various sources (e.g. Wormbase and KEGG).

#### Reactome

Reactome is a curated knowledgebase of biological pathways [29,30]. The goal of Reactome is to develop a curated resource of pathways and biochemical reactions in humans; however many of the reactions are also obtained via transfer from other species. The basic unit of this database is a reaction. Information on reactions is either derived from experiments in the literature or is an electronic inference based on sequence similarity. Reactions are also inferred in humans based on the putative human orthologs for the proteins that participate in the same reaction in other species. In such cases, the model organism reaction is annotated in Reactome, the inferred human reaction is annotated as a separate event, and the inferential link between the two reactions is explicitly noted. Each reaction is detailed with input, output, preceding and following events of the reaction, cellular component of the reaction and species of its occurrence. Each reaction is linked to pathways according to the order of reactions in corresponding pathway. The available pathways are integrated and represented graphically as a series of constellations in a 'starry sky.' This can be used to navigate through the reactions in biological pathways and visualize connections between them. It must be cautioned that the definition of PPIs in Reactome is quite broad: the interactions can be represented as 'direct complex,' 'indirect complex,' 'reaction' or 'neighboring reaction.' In a 'direct complex,' interactions occur between proteins present in the same complex and are not true pairwise interaction. 'Indirect complexes' contain interactions between interactors in different subcomplexes of a complex. 'Reactions' are interactions between proteins that participate in a reaction and the interactors are not reported to be in a complex. 'Neighboring reactions' represent the interactors that participate in 2 consecutive reactions, i.e. when one reaction produces a product, which is either an input or a catalyst for another reaction. The information is edited by the Reactome staff at Cold Spring Harbor Laboratory and the European Bioinformatics Institute and is then reviewed by other biological researchers for consistency and accuracy. Each reaction or pathway can be exported to Systems Biology Markup Language (SBML) and BioPAX formats. Reactome also provides tools such as Pathfinder and Skypainter. Pathfinder can identify pathways that connect input with output molecules while Skypainter allows the coloring of reaction maps based on user-specified identifiers that have been linked to each pathway. For our analysis, we have considered only the 'direct complexes' as they are the category most likely to correspond to true PPIs.

#### PDZBase

PDZBase is a database that focuses only on PPIs involving proteins with PDZ domains [31,32]. Only those interactions involving the PDZ domain that have been confirmed by individual *in vitro *or *in vivo *biochemical experiments are considered. Thus, interactions discovered solely through high-throughput methods (e.g. yeast two-hybrid or mass spectrometry) are not included in PDZBase. PDZ domains and their ligands can be queried using sequence motifs. Each interaction in PDZBase consists of the residues of the interacting proteins on a 2D-diagram generated by a residue-based-diagram-editor (RBDG). The interacting residues between the PDZ domain and their peptide ligands are predicted based on similarity with the available structures of PDZ-peptide complexes.

### Strategy used for comparison of datasets

The datasets were downloaded from the download sites of PPI databases on October 2, 2006 and scripts were used for parsing out the protein pairs involved in PPIs along with the experiment type and literature references, if provided. The PPIs were further parsed to extract binary interactions for those proteins pairs where both proteins were human. Most databases had Swiss-Prot as one of their accession identifiers except BIND which provided RefSeq, GenBank and PDB identifiers. To determine the overlap among databases, the Swiss-Prot or RefSeq identifiers were mapped to the corresponding Entrez Gene identifiers as of October 2, 2006. Scripts were used to convert these PPIs into a non-redundant list of PPIs (if protein A and B interact, the dataset may have two PPIs, A-B and B-A – only one of the PPI was retained for our analyses). All datasets were compared with each other to obtain the overlap at PPI and protein levels. Experiment types extracted for PPIs were mapped with PSI-MI vocabulary list. Disease annotations for genes were obtained from OMIM and mapped to gene symbols to obtain the number of proteins in PPIs corresponding to disease-associated genes.

### Caveats of comparing PPI data

Assessment of the accuracy of annotation of all PPIs in various publicly available databases is beyond the scope of this article. In this study, we have tried to evaluate parameters that could be measured objectively. Nevertheless, there are still a number of caveats of any analysis comparing PPIs. Below is a list of some of the potential pitfalls and our strategies to tackle them.

1. Binary interactions including homodimers were considered for this analysis while complex interactions were not. It is not easy to look at complex interactions across databases especially for comparison purposes although 'spoke' and 'matrix' models have been described previously for comparing protein complexes [33]. In this study, we have chosen not to compare the complex interactions because of predictive nature of these models. However, cases where a protein complex was already converted into binary PPIs by using one of these models (e.g. use of the 'matrix' model to computationally predict PPIs in Reactome) were treated as binary interactions.

2. Some of the binary interactions involved proteins that were non-human. Mapping of orthologs is not an easy task and is not standardized. Thus, we did not attempt to map the human orthologs for proteins from any other species that were listed as interacting proteins.

3. We mapped all protein isoforms to a unique gene and then examined the overlaps. This was done because often a given isoform is annotated as an interacting protein although the interaction is not specific to that isoform. For example, this strategy allowed us to correctly capture PPIs as overlapping where a given protein was annotated as interacting with one isoform of another protein in one database and with another isoform of that protein in another database.

## Results and Discussion

### Comparison of PPI data

**Figure 1:**
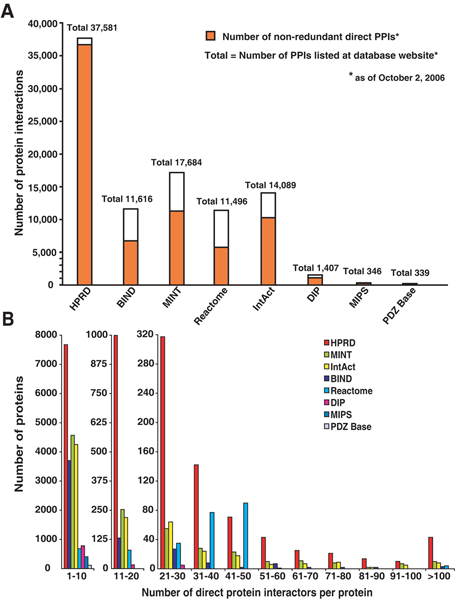
**Protein-protein interactions (PPIs) deposited in publicly available literature derived human PPI databases**. (*A*) Human PPIs present in interaction databases. The distribution of the number of PPIs annotated in each database is shown. For each bar, 'total' refers to the number of PPIs listed (i.e. claimed) at the database websites or number of PPIs in the downloaded datasets while the orange portion represents the number of human non-redundant direct PPIs calculated by us. (*B*) Distribution of the number of interacting proteins. Different scales are used to depict the number of proteins annotated with 1–10, 11–20, or 21–30 or higher number of PPIs per protein. All datasets were downloaded on October 2, 2006.

Table 1 summarizes the salient features of each database including total number of PPIs, total number of proteins, method of detection of PPIs, curation methodology, download options and URL links. The availability of data as a downloadable file is also indicated. Fig. 1A shows the distribution of the number of PPIs in each of the literature-based curated databases considered in our analysis. For each database, the total number of human PPIs present in the statistics page or in the downloaded files is shown along with the number of unique (non-redundant) binary human PPIs calculated by us. For this calculation, we only considered binary PPIs in which both members of an interacting pair were human proteins. As explained above, protein complexes were excluded from this analysis because it is difficult to ascertain the topology (i.e. which protein interacts with which protein in a complex) for determining overlap between datasets. The difference in the total and non-redundant PPIs in HPRD is because of protein complexes whereas in all other databases it is mainly due to the redundancy of PPIs. The distribution of PPI data in (Fig. 1A) shows a dramatic variation across these databases.

It is difficult to directly assess the depth of PPIs based on total interactions alone; thus, we analyzed the distribution of number of proteins in each database according to the number of binary (i.e. direct) interactions per protein. The majority of proteins in all databases have <10 interaction partners (Fig. 1B). The number of PPIs that fall under 31–40 and 41–50 PPI bins are high in HPRD and Reactome database. Although these PPIs are distributed across many types of proteins in HPRD, those in Reactome belong to mainly two classes: proteosomal or ribosomal protein complexes. The number of interactions for these two classes of proteins in Reactome is high because a 'matrix' model of interpreting protein complexes is used in which all proteins are considered connected to all proteins within a complex. All other database shows the same trend with a greater number of proteins in bins with lower number of PPIs per protein. This does not automatically imply that most proteins truly interact with a small number of interactors. Rather, this is likely due to the fact that not all proteins have been studied thoroughly and because all published interactions have not yet been included in these databases. Additionally, there is a bias of experimental methods in capturing all interactions (e.g. yeast two-hybrid system does not generally detect interactions involving integral membrane proteins). Overall, most databases contain a very small number of proteins with >30 PPIs.

### Comparison of proteins annotated with PPIs

**Figure 2:**
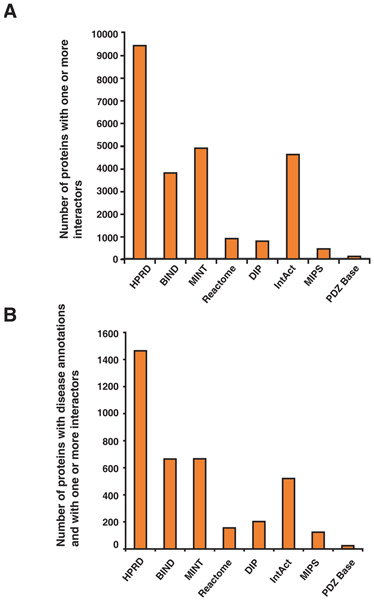
**Protein coverage across human PPI databases**. (A) The total number of non-redundant genes whose protein products are annotated in the databases with at least one PPI. (B) The number of proteins encoded by human disease-associated genes listed in OMIM database with at least one PPI.

We looked for the total number of unique genes represented in the PPI databases (Fig. 2A). In HPRD, proteins encoded by 9,427 genes have at least one or more direct PPI annotated (out of ~20,000 proteins annotated in this database) while BIND, IntAct and MINT contain 3,887, 4,614 and 4,975 proteins, respectively. Other databases such as DIP, Reactome, MIPS and PDZ Base contain PPIs for <1000 proteins.

### Proteins encoded by disease-associated genes in PPIs

PPIs are attractive as potential targets for small-molecule drugs for treatment of diseases. We checked for proteins encoded by genes listed in the OMIM database that are mutated in inherited genetic disorders (Fig. 2B). HPRD has all human disease-associated genes listed in OMIM of which 1,463 have at least one protein interactor while most of the other databases contain significantly less number of proteins encoded by these genes.

### Overlap of PPIs and proteins between databases

**Figure 3:**
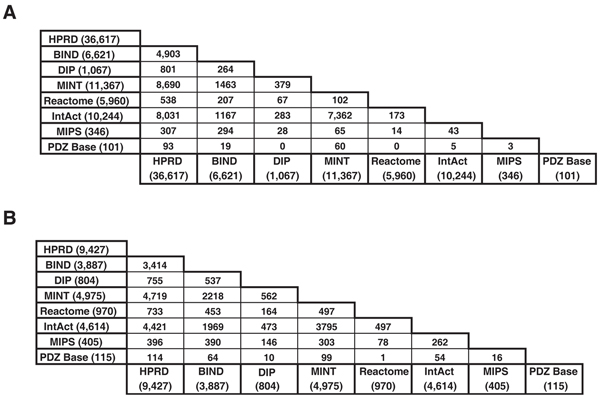
**Overlap of PPIs and proteins in human PPI databases**. (*A*) Pairwise overlap of protein interactions across databases is shown in cells. The number of non-redundant direct PPIs present in each database is shown in parentheses for each database. (*B*) Pairwise overlap of proteins across databases is shown in the cells. The number of non-redundant proteins present in each database is shown in parenthesis for each database.

As discussed above, there is a significant difference in the total number of PPIs in the various databases. However, this statistic does not provide an idea of the extent to which the PPIs actually overlap across databases. As shown in Fig. 3A, HPRD contains a high proportion of human PPIs that are present in other literature-derived curated databases. The overlap between IntAct (10,244 PPIs) and MINT (11,367 PPIs) is 7,362, which is the highest overlap among the remaining literature-derived databases; the overlap between BIND (6,621 PPIs) and MINT (11,367 PPIs) is only 1,463 and there is no overlap between PDZBase and DIP.

To determine whether the overlap is small because of proteins not being annotated in different databases, we looked at the overlap at the protein level between databases. As shown in Fig. 3B, the overlap of proteins between BIND (3,887 proteins) and IntAct (4,614 proteins) is 1,969 but the overlap at PPI level is only 1,167. HPRD contains 76% and MINT contains 51% of proteins in Reactome, although there is a very low overlap at the level of PPIs across these databases. Overall, although at protein level there is a good overlap between the databases, the PPIs do not overlap as much. Average degree (K) of a protein i.e. the number of interactions that a protein has with other proteins, is 7.6 for HPRD, while that for MIPS, PDZ Base, DIP, BIND, MINT and IntAct ranges from 1.7 to 4.5. Strikingly, the average degree of a protein in Reactome is 12.2, which is because of the interpretation of protein complexes through the 'matrix' model as explained above.

We also carried out a comparison of a test set of proteins to check the distribution of interaction partners of PPIs across different databases (Table 2). The test proteins were selected based on the presence of proteins in four or more databases. We required that the protein be present in four or more databases because there was not even a single protein that was common to all databases. The proteins were further selected to cover proteins that participate in several different types of biological processes to avoid any potential bias in the event that any particular database is especially 'strong' in certain types of annotations. As shown in Table 2, Caspase 3 (CASP3) has 126 protein interaction partners annotated in HPRD, while BIND, MINT, IntAct and Reactome contain 15, 6, 3 and 1 interaction, respectively. S-phase kinase-associated protein 1A (SKP1A) has 35 PPIs in HPRD, 11 in BIND, 5 in DIP and 13 in MINT. MIPS and PDZBase do not contain any PPIs for this protein. Nuclear factor kappa-B subunit 3 (RELA) has 98 protein interaction partners in HPRD while BIND, MINT, DIP and IntAct contain 13, 103, 13 and 90 PPIs. Overall, for most proteins, there is at least one, and often several, databases that do not contain any PPI annotations (Table 2). This again reflects the fact that the databases are still at an early stage of curation and annotation of published PPIs.

### Literature citations in literature-derived databases

**Figure 4:**
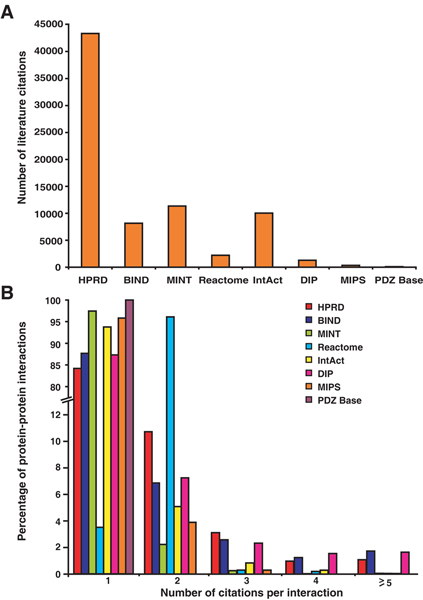
**Literature citations for protein-protein interactions**. (*A*) The total number of literature citations linked to PPIs. (*B*) The percentage of PPIs in databases corresponding to 1, 2, 3, 4, or ≥5 literature citations per interaction is shown. The scale is modified as shown to provide a better view of the distribution of proteins with two or more citations per interaction.

Literature citations are generally linked to interactions in literature-derived datasets. We checked the total citations in PubMed linked to PPIs in the literature-derived databases (Fig. 4A). HPRD has >43,634 published articles to support the PPI data, while BIND and MINT contain ~8,020 and ~11,480 citations, respectively. Reactome contains a total of ~2,000 citations. Another parameter to assess the extent of curation is to determine the number of citations per interaction. More than one citation for a given PPI indicates that the interaction has been verified by more than one group or method. Conversely, however, the presence of a single citation does not automatically imply that there is only one study describing the interaction because it is quite likely that only one published paper was linked although several studies might have been carried out (i.e. incomplete curation). This is illustrated in the section below where the same PPI is compared across multiple databases. As shown in Fig. 4B, 100% of PPIs in PDZBase and >95% of PPIs in MINT, IntAct and MIPS had one PubMed citation. In contrast, 87% in BIND and DIP and 84% of PPIs in HPRD have only one citation. Notably, ~11% and 7% of PPIs in HPRD and BIND, respectively, have 2 citations and ~2% of PPIs in HPRD, BIND and IntAct have more than 5 citations each. The majority of PPIs in Reactome (~96%) are linked to the same 2 published articles because these PPIs are predicted computationally using a matrix approach (i.e. all against all) to link proteins that were identified in two mass spectrometry-based protein complex pulldown studies on spliceosomes [34,35].

### Comparison of PPI annotations common to multiple databases

Overall statistics of databases might not reflect the breadth and depth of protein annotations from a biologist's perspective. To provide certain 'case studies,' we prepared a list of protein interactions that are common to 4 or more literature-derived databases and then tabulated the number of PPIs in each database. We left out PDZBase because of its small size. Table 3 lists 6 representative PPIs that were common to 4 or more databases along with the article(s) cited for each interaction and the annotation of the experimental methods used to detect the corresponding PPI. As an example, the experimental method annotated for the interaction between transcription factors NFKB1 and NFKB3 reported recently [36] is *in vivo *(MI:0492) in HPRD, tandem affinity purification (TAP) (MI:0045) in DIP, anti tag coimmunoprecipitation (MI:0109) in MINT and tap tag coip (MI:0007) in IntAct. This example illustrates how databases can describe the same experiment using alternative vocabulary terms. The interaction, TNFRSF1A with TRADD, is annotated as *in vivo, in vitro *and yeast 2-hybrid with 3 PubMed citations in HPRD, simply 'experimental' with 1 PubMed citation in DIP, immunoprecipitation and affinity chromatography with 3 PubMed citations in BIND, co-immunoprecipitation with 1 PubMed citation by MIPS, 'co-immunoprecipitation, pulldown and two hybrid' with 2 citations by MINT and 'anti-bait coip, pulldown and two hybrid' with 1 citation by IntAct. Together, the 6 databases refer to 8 PubMed citations to describe this interaction while each individual database only uses between 1 and 3 citations. For the interaction of FADD with FAS, HPRD annotation is '*in vivo*, *in vitro *and yeast 2-hybrid,' DIP mentions 'two hybrid test,' BIND describes it as 'immunoprecipitation', MIPS mentions 'coip,' MINT describes it as 'coimmunoprecipitation and two hybrid' and IntAct annotates it as coip, pull down, anti tag coip and two hybrid.' Table 3 highlights how different databases use different published articles for annotating the same PPI. Thus, mere presence of a PPI in different literature-derived databases does not automatically guarantee that the annotations will be identical. It also illustrates that merging of annotations from multiple databases will lead to an increase in the depth of individual annotations.

### Download options and use of identifiers in PPI databases

Proteomics Standards Initiative (PSI) is a collaborative initiative for standardization of protein-related data including protein-protein interaction and mass spectrometry data. PSI-molecular interaction (PSI-MI) [37] format is an exchange format, which has already become the standard for PPI data [4]. Table 1 shows that although many databases provide the PPI data in this format such as HPRD, BIND, DIP MINT, MIPS and IntAct, some databases such as AfCS and Reactome do not currently have this option. Reactome also provides data in two pathway-related formats, BioPAX and SBML. The data contained in AfCS is not currently available as a downloadable file.

Although a consensus on the use of standardized vocabulary for denoting PPIs is evolving and is being increasingly used, there is no requirement for use of any particular type of identifiers or database accession numbers for proteins in PPI databases. Different sets of protein database identifiers are used, with many of them being frequently retired, merged or otherwise updated. This creates great difficulties for those who want to combine datasets from different databases. It is not a trivial task to 'map' identifiers to a single set of proteins and creates a bioinformatics pitfall of its own. If this 'mapping' is done by purely automated methods, there is a risk of wrong assignment of a protein entry from one database to another. To minimize this, we recommend the use of gene symbols in addition to any 'favorite' protein identifier. This allows for a relatively more error-free interpretation of PPI data at the gene level.

## Conclusion

There is great interest in protein-protein interactions as a means of understanding the complexities of a cell. Large scale PPI data derived from high-throughput experiments or literature derived curated databases has been used to analyze the molecular networks of human cells [38-41]. Here, our assessment shows that the number of PPIs in databases varies widely from as low as 100 to over 36,600 interactions. Overlap of PPIs within the same category of databases (e.g. within literature-derived databases) is low despite the presence of overlapping proteins. A comparison of the number of PPIs for a test set of proteins confirms that there is indeed a large variation in the number of interactors across the interaction databases. Also, a comparison of annotations for the PPIs that do overlap between the databases reveals differences in annotations through the use of alternative vocabulary terms. This is partly because of the difference in interpretation of the experimental results by the biologists annotating them and partly because of the overlapping meaning of the terms themselves.

A particularly important issue is that of protein isoforms. Often, only one isoform is annotated as an interactor although there is no evidence that the interaction is specific to that isoform. In other experiments such as coimmunoprecipitation experiments, it is almost impossible to discern which isoform binds unless an isoform-specific antibody is used. Because of this difficulty in mapping isoforms, we suggest that groups carrying out interaction studies, especially large-scale studies, map the identity of the proteins to genes and include this in their data submission. We have also previously done this for protein identification studies using mass spectrometry where a similar difficulty exists with regard to identification of particular isoforms [42]. If this is done, then a binary interaction can be interpreted thus: at least one of the gene products of Gene A interacts with at least one of the gene products of Gene B.

The dissemination of PPI datasets is an important aspect for optimal use of the data. Through decades of research, molecular biologists have discovered a large number of PPIs. Collecting this information, storing it and maintaining a database is a valuable task, which is perhaps not adequately appreciated by the scientific community. Our evaluation of human PPI databases highlights the diverse nature of annotation and representation of PPIs in databases. We hope that this review will assist biomedical scientists in making informed decisions about the most appropriate database to suit their needs and to actively participate with the databases to maintain error-free and updated annotations.

## List of Abbreviations

PSI-MI: Proteomics Standards Initiative – Molecular Interaction

HPRD: Human Protein Reference Database

BIND: Biomolecular Interaction Network Database

DIP: Database of Interacting Proteins

MINT: Molecular INTeraction database

AfCS: Alliance for Cellular Signaling

## Authors' contributions

SM and AP conceived the study design, SM, BP, TKBG, KK and SS carried out the data mining work and SM, TKBG and AP drafted the manuscript and RM and YLR provided comments. All authors read and approved the final manuscript.

## Figures and Tables

**Table 1:** Unique features of human PPI databases

	**Number of unique human PPIs**	**Number of proteins**	**PPI data**	**Unique features**	**Download options**	**PSI-MI compatibility**	**Download version number**
**HPRD**	36,617	9,427	Experimental	Protein annotations are included (e.g. PTMs, substrate information, tissue expression, disease association, protein complexes, subcellular localization). Signal transduction pathways	Yes	Yes	Release 6
**BIND**	6,621	3,887	Experimental	Protein complexes, biological pathways, non-protein interactions, Data for >1473 organisms	Yes	Yes	20060525
**DIP**	1,067	804	Experimental	PPIs for other organisms, protein complexes	Yes	Yes	Hsapi20060402
**MINT**	11,367	4,975	Experimental	PPIs for other organisms, non-protein interactions	Yes	Yes	Version 18
**PDZBase**	101	115	Experimental	PPIs involving PDZ domains. Prediction of residues that interact.	No	No	October 2, 2006
**MIPS**	346	405	Experimental	PPIs for other organisms	Yes	Yes	October 2, 2006
**IntAct**	10,244	4,614	Experimental	Protein complexes, PPIs for other organisms, non-protein interactions, provides web based applications, ProViz and Hierarch View, for visualization of interactions	Yes	Yes	2006-09-22
**AfCS**	Mostly mouse interactions	Mostly mouse proteins	Experimental	Protein annotations are included (e.g. function, subcellular localization, orthologs, tissue expression, mouse knockout phenotype information, PTMs)	No	No	-
**REACTOME**	5,960	970	Experimental, automated and predicted	Biological pathways for several organisms. Navigation through reactions in biological pathways and visualizing connections between them	Yes	No	Version 18

**Table 2:** Comparison of protein-protein interactions for a test set of proteins.

	**HPRD**	**BIND**	**DIP**	**MINT**	**IntAct**	**MIPS**	**PDZBase**	**Reactome**
**CASP3**	126	15	0	6	3	0	0	1
**CDK2**	71	16	9	11	12	2	0	2
**TBP**	81	17	14	12	15	2	0	14
**TNFRSF1A**	43	11	8	77	74	1	0	1
**YWHAB**	116	12	4	83	6	1	0	2
**GAPDH**	37	6	0	20	19	0	0	0
**RELA**	98	13	13	103	90	1	0	2
**HDAC1**	114	13	5	14	12	1	0	0
**RPS27**	2	1	0	9	10	0	0	32
**SKP1A**	35	11	5	13	15	0	0	2
**ACTC**	32	2	0	1	2	0	0	0
**PABPC1**	23	3	0	11	6	0	0	2
**VDAC1**	16	4	0	2	0	2	0	0
**THRB**	35	11	0	0	2	2	0	0
**HSPA8**	42	5	0	42	40	0	0	0
**PDZK1**	17	0	0	3	4	0	1	0

**Table 3:** Comparison of annotations of PPIs common to literature-derived curated PPI databases

	**Interacting Proteins**		**HPRD**		**DIP**		**BIND**		**MIPS**		**MINT**		**IntAct**	
			**Detection method**	**PubMed ID**	**Detection method**	**PubMed ID**	**Detection method**	**PubMed ID**	**Detection method**	**PubMed ID**	**Detection method**	**PubMed ID**	**Detection method**	**PubMed ID**

1	NFKB1	NFKB3	*in vivo*	9101089	Tandem Affinity Purification (TAP)	14743216	Gel retardation assays, three dimensional structure	15735750, 9738011, 9865693	-	-	anti tag coimmuno- precipitation	14743216	Comigration in gel, anti bait coip, tap	8246997, 8246997, 14743216
2	TNFRSF1A	TRADD	*in vivo, in vitro, Yeast 2-hybrid*	7758105, 8565075, 8612133	Experimental	9129204	Immuno- precipitation	11684708, 15247912, 9916731	coip: coimmuno precipitation	9916731	Coimmuno- precipitation, pull down, two hybrid	8565075, 8621670	anti bait coip, pull down, two hybrid	7758105
3	FADD	FAS	*in vivo, in vitro*, Yeast 2-hybrid	8967952, 7538907, 7536190	Two hybrid test	7538907	Immuno- precipitation	15665818, 15383280	coip: coimmuno precipitation	10196099	Coimmuno-precipitation, two hybrid	7536190, 7538907	anti tag coip, coip, pull down, two hybrid	7538907, 7536190, 7538907, 7538907
4	PEX19	PEX3	*in vivo, in vitro, Yeast 2-hybrid*	10704444, 12096124	-	-	two-hybrid-test	10430017, 12096124	coip: coimmuno precipitation, two hybrid	10430017	two hybrid, ubiquitin reconstruction	12096124, 16189514	far western blotting, two hybrid pooling	10704444, 16189514
5	CDK2	CDKN1A	*in vitro*	12839982	Two hybrid test	8242751	other	15232106	coip: coimmuno precipitation	8641969	protein array, pull down	15232106, 9284049	protein array, pull down	15232106, 8756624
6	PEX12	PEX5	*in vivo, in vitro*, Yeast 2-hybrid	10562279, 10837480, 12096124	-	-	two-hybrid-test	12096124	coip: coimmuno precipitation, two hybrid	10646847	two hybrid	12096124	anti tag coip, filter binding, two hybrid	10562279, 10562279, 12620231
